# Microvirus Genomes Identified in Fecal Samples from Yellow-Bellied Marmots

**DOI:** 10.1128/mra.01218-21

**Published:** 2022-02-10

**Authors:** Cristal L. Tijerino, Daniel T. Blumstein, Simona Kraberger, Arvind Varsani

**Affiliations:** a Biodesign Center for Fundamental and Applied Microbiomics, School of Life Sciences, Center for Evolution and Medicine, Arizona State University, Tempe, Arizona, USA; b Department of Ecology and Evolutionary Biology, University of California, Los Angeles, Los Angeles, California, USA; c Structural Biology Research Unit, Department of Integrative Biomedical Sciences, University of Cape Town, Cape Town, South Africa; DOE Joint Genome Institute

## Abstract

The complete genomes of 63 unique microviruses were determined from fecal samples collected from three yellow-bellied marmots living in Colorado, USA. These microviruses are diverse, with genome sizes ranging from 4,265 to 7,225 nucleotides, and have varied presence across the three samples.

## ANNOUNCEMENT

Yellow-bellied marmots (*Marmota flaviventrus*) are semifossorial, herbivorous sciurid rodents native to the mountain and intermountain regions in western regions of North America (1, 2). There is limited information on viruses associated with marmots in general, and studies have reported rabies virus in groundhogs (3), bocaparvovirus in Himalayan marmots (4), and California encephalitis virus (5), anelloviruses, genomoviruses, and various unclassified cressdnaviruses in yellow-bellied marmots (6). Here, we build on our previous virus work on yellow-bellied marmots (6) with a focus on bacteriophages.

Fecal samples from three yellow-bellied marmots (marmot identification numbers 8189-7849 [Mar1], 7828-8390 [Mar2], and 8265-6223 [Mar3]) living in the upper East River Valley, in and around the Rocky Mountain Biological Laboratory (near Crested Butte, CO, USA), were collected on 18, 21, and 22 May 2018. The fecal samples were processed as outlined by Steel et al. (7). Viral DNA was extracted using the High Pure viral nucleic acid kit (Roche Diagnostics, USA), and circular DNA was amplified using rolling circle amplification (RCA) with a TempliPhi 2000 kit (GE Healthcare, USA). The resulting RCA DNA was used to prepare 2 ×100-bp libraries with the Nextera DNA library preparation kit, and the libraries were sequenced in an Illumina HiSeq 4000 sequencer. All bioinformatic tools used were run with default parameters. Trimmomatic v0.39 (8) was used to trim the raw reads, which were then *de novo* assembled with metaSPAdes v3.12.0 (9). Bacteriophage-like sequences were identified using VirSorter (10). Genomes of 63 unique bacteriophages (determined to be circular on the basis of terminal redundancy) were identified among the three samples, with a genome size range of 4,265 to 7,225 nucleotides (nt). RASTtk (11) was used to determine the open reading frames, which were annotated on the basis of BLASTp (12) similarities to proteins encoded by microvirus sequences available in GenBank.

**FIG 1:**
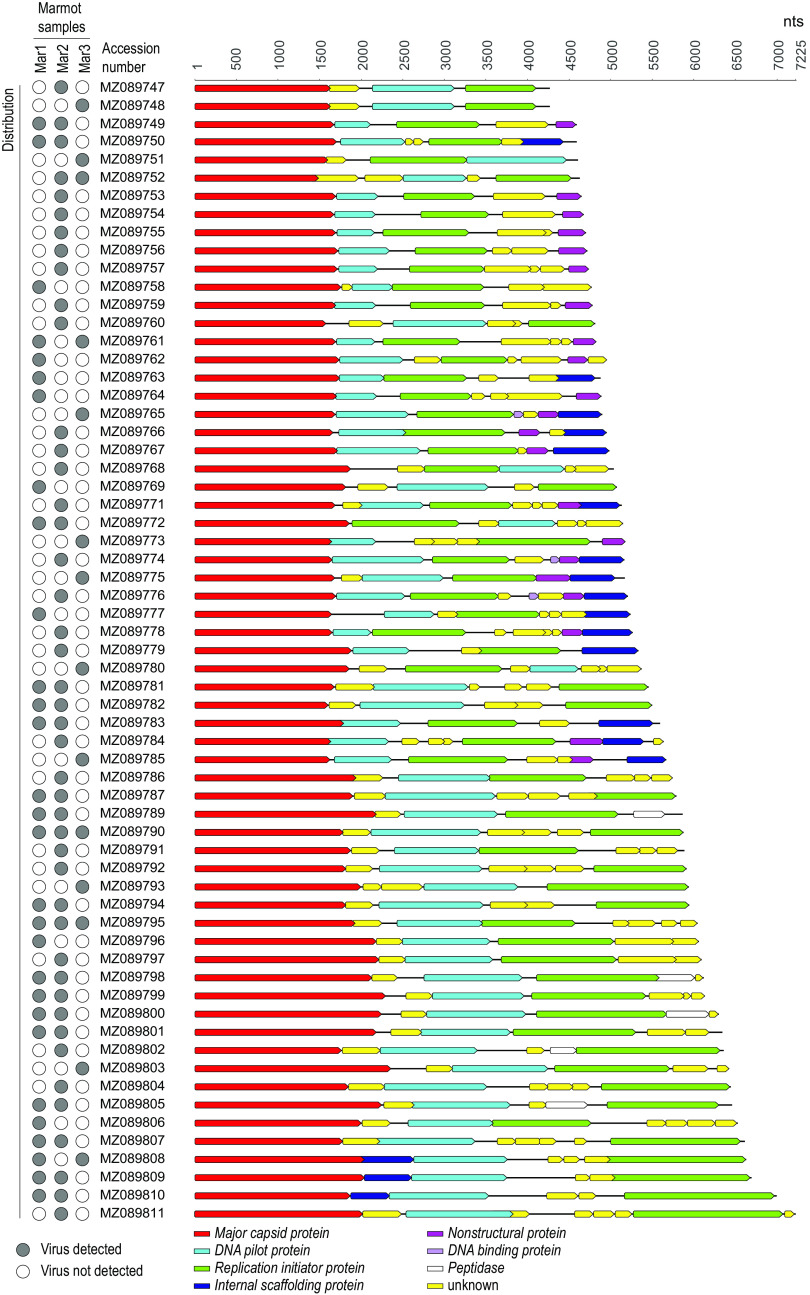
Distribution and genome organization of the 63 microvirus genome sequences across the three individual samples (Mar1, identification number 8189-7849; Mar2, identification number 7828-8390; Mar3, identification number 8265-6223). Solid gray circles indicate 100% raw read genome coverage and thus the presence of the virus sequence in the sample.

Since we were able to identify many of the bacteriophage genomes in more than one sample, we used the raw reads from each individual library to map to the unique genomes (Fig. 1), using BBMap to investigate their presence across the three samples (13). Based on BLASTx analysis against a viral protein RefSeq database, these 63 bacteriophage genomes were determined to be part of the family *Microviridae* and likely the subfamily *Gokushovirinae*. Microviruses are small circular single-stranded DNA viruses with T=1 icosahedral capsids (14, 15) in the order *Petitvirales* and phylum *Phixviricota*. The marmot-feces-derived microviruses have GC contents of 33.10 to 51.70% (Table 1) and encode at least a major capsid protein, DNA pilot protein, and replication initiator protein (Fig. 1). The genomes have read depths of 13.8× to 51,700.9×, and the numbers of mapped reads ranged from 686 to 2,733,933 reads (Table 1). The unique microviruses have variable distribution in the three marmot fecal samples, with only two being present in all three samples (Fig. 1). It is likely that these microviruses infect enterobacteria that are part of the microbial gut flora of yellow-bellied marmots. The major capsid proteins of the 63 microviruses share >27% amino acid identity with all of the complete genomes available in GenBank (downloaded on 24 October 2021).

**TABLE 1:** Summary of findings for the microviruses identified in this study

GenBank accession no.	Marmot-feces-derived microvirus	Genome length (nt)	GC content (%)	Coverage depth (×)	No. of reads	Top BLASTp hit for major capsid protein		
						GenBank accession no.	Microvirus	Pairwise identity (%)
MZ089747	Microvirus mar1	4,265	40.70	326.8556	13,843	MZ089748	Microvirus mar2	96.10
MZ089748	Microvirus mar2	4,265	40.80	598.3988	25,317	MZ089747	Microvirus mar1	96.10
MZ089749	Microvirus mar3	4,586	42.80	316.6334	14,443	MZ089754	Microvirus mar8	70.80
MZ089750	Microvirus mar4	4,589	47.90	192.8311	8,804	MT185428	Escherichia phage EC6098	84.10
MZ089751	Microvirus mar5	4,605	35.30	29,622.12	1,355,967	MT309922	*Microvirus* sp. strain BS1_560	44.00
MZ089752	Microvirus mar6	4,626	45.90	568.695	26,155	MH992222	Apis mellifera-associated microvirus 54	37.40
MZ089753	Microvirus mar7	4,646	45.60	359.5489	16,605	MZ089759	Microvirus mar13	70.10
MZ089754	Microvirus mar8	4,671	41.30	54.7116	2,541	MZ089749	Microvirus mar3	70.80
MZ089755	Microvirus mar9	4,696	37.60	380.3922	17,799	MZ089754	Microvirus mar8	63.90
MZ089756	Microvirus mar10	4,713	34.50	293.1483	13,690	MZ089757	Microvirus mar11	52.80
MZ089757	Microvirus mar11	4,725	38.60	156.9342	7,371	MZ089754	Microvirus mar8	65.40
MZ089758	Microvirus mar12	4,765	47.50	9,341.269	442,289	MZ089763	Microvirus mar17	43.70
MZ089759	Microvirus mar13	4,777	43.20	523.0358	24,834	MZ089753	Microvirus mar7	70.10
MZ089760	Microvirus mar14	4,811	45.50	905.264	43,305	MG945631	*Microviridae* sp. strain 6228-1612	31.90
MZ089761	Microvirus mar15	4,815	39.80	5,850.599	279,887	MZ089749	Microvirus mar3	64.00
MZ089762	Microvirus mar16	4,862	44.40	4,021.423	194,203	MG945484	*Microviridae* sp. strain 870-1801	56.50
MZ089763	Microvirus mar17	4,873	38.40	471.1172	22,804	MG945416	*Microviridae* sp. strain 4186-1801	46.60
MZ089764	Microvirus mar18	4,882	46.40	14.1657	686	MZ089757	Microvirus mar11	49.60
MZ089765	Microvirus mar19	4,892	44.00	50.409	2,454	MG945793	*Microviridae* sp. strain 4431-1801	75.50
MZ089766	Microvirus mar20	4,946	43.00	55.6852	2,741	MG945530	*Microviridae* sp. strain 2245-1801	75.90
MZ089767	Microvirus mar21	4,978	45.80	32.811	1,628	MG945602	*Microviridae* sp. strain 4458-1602	74.30
MZ089768	Microvirus mar22	5,030	37.90	105.1563	5,264	MH572523	*Microviridae* sp. strain SD_HF_35	35.80
MZ089769	Microvirus mar23	5,069	43.50	143.7889	7,241	KM589515	*Parabacteroides* phage YZ-2015a	41.00
MZ089771	Microvirus mar25	5,129	40.40	90.2977	4,532	MG945783	*Microviridae* sp. strain 4215-1801	54.90
MZ089772	Microvirus mar26	5,144	36.60	86.928	4,391	MZ089780	Microvirus mar34	64.24
MZ089773	Microvirus mar27	5,158	41.40	1,514.159	77,508	KX513866	Human gut gokushovirus SH-CHD3	48.20
MZ089774	Microvirus mar28	5,162	44.90	203.4876	10,423	MG945158	*Microviridae* sp. strain 381-1801	68.40
MZ089775	Microvirus mar29	5,167	51.70	469.8905	24,153	MG945541	*Microviridae* sp. strain 2464-1801	77.10
MZ089776	Microvirus mar30	5,204	37.40	477.1973	24,651	KR704914	Chimpanzee-feces-associated microphage 2	68.70
MZ089777	Microvirus mar31	5,235	42.90	272.6846	14,186	MG945231	*Microviridae* sp. strain 1313-1801	41.80
MZ089778	Microvirus mar32	5,259	41.30	19.8427	1,036	MZ089775	Microvirus mar29	45.70
MZ089779	Microvirus mar33	5,328	49.90	51,700.9	2,733,933	KT264795	Gokushovirus WZ-2015a strain 47Fra09	41.80
MZ089780	Microvirus mar34	5,368	37.90	13.8117	737	MZ089772	Microvirus mar26	64.30
MZ089781	Microvirus mar35	5,453	43.60	30,407.66	1,646,981	MG945631	*Microviridae* sp. strain 6228-1612	30.40
MZ089782	Microvirus mar36	5,497	45.10	4,459.733	243,249	MW149098	*Microvirus* sp. strain gila10	33.00
MZ089783	Microvirus mar37	5,589	33.10	529.9927	29,408	MH617213	*Microviridae* sp. strain ctia17	41.50
MZ089784	Microvirus mar38	5,634	34.20	15.8601	888	KT264826	Gokushovirus WZ-2015a strain 78Fra08	53.70
MZ089785	Microvirus mar39	5,664	36.80	127.0998	6,975	MK496790	Capybara microvirus Cap3_SP_441	73.70
MZ089786	Microvirus mar40	5,741	37.40	318.3928	17,056	MZ089795	Microvirus mar49	71.10
MZ089787	Microvirus mar41	5,785	42.70	357.0657	20,406	MW149098	*Microviridae* sp. strain gila10	38.63
MZ089789	Microvirus mar43	5,863	38.50	624.3561	36,264	MH552492	*Microviridae* sp. strain ctbc205	58.70
MZ089790	Microvirus mar44	5,873	42.90	2,807.073	163,702	MZ089794	Microvirus mar48	51.10
MZ089791	Microvirus mar45	5,881	39.60	344.7458	19,361	MZ089795	Microvirus mar49	71.20
MZ089792	Microvirus mar46	5,909	40.20	2,029.845	119,011	MZ089794	Microvirus mar48	54.50
MZ089793	Microvirus mar47	5,933	40.40	182.7396	10,764	MW149103	*Microvirus* sp. strain gila14	45.00
MZ089794	Microvirus mar48	5,939	40.40	4,102.556	241,928	MZ089792	Microvirus mar46	53.90
MZ089795	Microvirus mar49	6,039	46.50	4,253.266	254,701	MZ089786	Microvirus mar40	71.10
MZ089796	Microvirus mar50	6,058	41.50	829.5149	49,720	MZ089797	Microvirus mar51	81.10
MZ089797	Microvirus mar51	6,091	41.80	54.4912	3,290	MZ089796	Microvirus mar50	81.10
MZ089798	Microvirus mar52	6,115	40.10	3,366.664	203,191	MZ089796	Microvirus mar50	47.20
MZ089799	Microvirus mar53	6,128	42.20	1,465.885	89,140	MZ089803	Microvirus mar57	70.60
MZ089800	Microvirus mar54	6,294	41.80	81.4083	5,086	MG945616	*Microviridae* sp. strain 5104-1801	42.90
MZ089801	Microvirus mar55	6,340	43.30	381.0931	23,982	MW149104	*Microvirus* sp. strain gila3	50.90
MZ089802	Microvirus mar56	6,357	38.80	3,515.274	221,386	MT310368	*Microvirus* sp. strain 1712115_272	61.00
MZ089803	Microvirus mar57	6,419	42.60	1,8670.08	1,189,287	MZ089799	Microvirus mar53	70.60
MZ089804	Microvirus mar58	6,439	43.60	478.1224	30,606	MZ089807	Microvirus mar61	53.10
MZ089805	Microvirus mar59	6,455	37.70	1,046.683	66,856	MW149104	*Microvirus* sp. strain gila3	49.70
MZ089806	Microvirus mar60	6,523	34.70	235.7892	15,164	MH552552	*Microviridae* sp. strain ctid22	46.70
MZ089807	Microvirus mar61	6,609	42.90	3,545.174	231,859	MZ089804	Microvirus mar58	54.90
MZ089808	Microvirus mar62	6,623	43.50	12,365.8	813,167	MZ089809	Microvirus mar63	63.90
MZ089809	Microvirus mar63	6,688	48.10	2,319.365	153,964	MZ089808	Microvirus mar62	63.90
MZ089810	Microvirus mar64	6,994	48.30	71.5102	4,920	MH552557	*Microviridae* sp. strain ctbd329	54.30
MZ089811	Microvirus mar65	7,225	38.40	325.8725	23,322	MT310367	*Microvirus* sp. strain 1712115_282	27.60

### Data availability.

The sequences of the microviruses have been deposited in the NCBI databases under BioProject accession number PRJNA722789 (SRA accession numbers SRX10636081, SRX10636082, and SRX10636083) and GenBank accession numbers MZ089747 to MZ089769, MZ089771 to MZ089797, and MZ089799 to MZ089811.
